# Ten simple rules for defining a computational biology project

**DOI:** 10.1371/journal.pcbi.1010786

**Published:** 2023-01-05

**Authors:** William Stafford Noble

**Affiliations:** 1 Department of Genome Sciences, University of Washington, Seattle, Washington, United States of America; 2 Paul G. Allen School of Computer Science and Engineering, University of Washington, Seattle, Washington, United States of America; Dassault Systemes BIOVIA, UNITED STATES

This is a *PLOS Computational Biology* Methods paper.

## Introduction

If you are working in the field of computational biology, then hopefully you are familiar with the excitement associated with coming up with a new idea and thinking about how to follow up on it. Maybe the idea came from a talk you heard at a conference, a paper you read, or a conversation with a colleague. Regardless, your brain is now abuzz with how this idea will be implemented and what data you’ll need to validate it. Ultimately, if your idea pans out, perhaps it will lead to profound scientific insights, a high-impact paper, and a widely used software tool. But for now, it’s just an idea in your head. How do you begin to bring your new idea to fruition? This is, of course, the core of the scientific method–transforming an idea (or hypothesis) into discoveries. Hence, your success as a scientist depends strongly on your ability to efficiently and effectively carry out such transformations.

Here, I focus on the very first few steps of that transformation, providing some general rules that can help start your new project in the right direction. Some of these rules may be considered variations on the questions asked in the Heilmeier catechism (https://www.darpa.mil/work-with-us/heilmeier-catechism), which is a set of questions that the US Defense Advanced Projects Agency uses to evaluate potential research projects.

### Rule 1: Write it down

Many computationally inclined people prefer writing computer code to writing English. Unfortunately, if you get excited by an idea and immediately dive into implementing and validating it, then you can end up pursuing a half-baked idea that, on second thought, really doesn’t make sense. The act of writing your idea down will inevitably clarify it for you. I typically think of this writeup as a first draft of the introduction of the paper you eventually aim to write. Rules 2 to 8 describe this writeup in more detail.

### Rule 2: Define the problem you want to solve

It is important up front to clearly delineate the scope and precise nature of the problem your work will address. If you are proposing a novel computational method, you will want to specify precisely what types of inputs are expected and what the resulting output will be.

### Rule 3: Define the potential impact

Are you aiming to solve an important problem? You should think carefully about who would benefit from a solution to this problem and what those benefits will be. The more clearly you can spell out the potential impacts of your work, the better you’ll be able to motivate your project when you describe it to your friends, colleagues, funding agencies, and eventually to the readers of your paper. Thinking about impact can also help to focus (or refocus) your work.

### Rule 4: Summarize the related work

A terrible trap to fall into is carrying out an entire research project only to find that the same work was already published before you began. Avoid this trap by doing your literature review up front. Also, reading the literature has the (hopefully obvious) added benefit that other people’s work can provide you with good ideas. It is important, early on, to identify all of the relevant work and at least summarize it at a high level. I typically like to do this by taking some notes on each method and then, in the overview document, summarizing methods in a chronologically ordered table, where rows are papers and columns are features of each method or vice versa. Here are some examples: [1–3].

### Rule 5: State your central hypothesis

Boiling down your idea to a single sentence can be a powerful way to achieve focus and ensure that your project does not wander. Of course, you may have multiple, related ideas, but I find it helpful to try to coalesce these into a single, overarching theme. This will be easier to do if you recognize up front that the hypothesis itself might change during the course of the project. Thus, it will be worthwhile to periodically revisit your stated hypothesis and ask yourself whether it is still the driving idea behind your research.

### Rule 6: Sketch out your approach

I am not suggesting writing a detailed methods section for your paper at this stage, but I do think it’s worthwhile to write out in English the basic idea behind the computational method you plan to implement. Writing this description will make the overview document self-contained and can also help clarify what the approach consists of prior to writing the code itself. An important part of this sketch involves identifying the pros and cons of your proposed approach compared with available alternatives. This might involve, for example, relative speed, accuracy in certain situations, or scaling to a large number of dimensions. In general, you should be clear about the potential strengths and weaknesses of your approach.

### Rule 7: Identify available data

While in principle, it is possible to carry out an entire computational biology project without ever looking at real data, such projects are rare. In most cases, you will be constrained to analyze data that exists or data that you (or your collaborators) can produce for the project. In particular, it’s important to be sure that you not only have the input data, but also the expected outputs; i.e., you need to have some way to demonstrate that your method works as expected. Keep in mind that simulated data can often be useful, sometimes as a way to validate that your model works under an explicit set of assumptions or to test specific hypotheses.

### Rule 8: Choose appropriate validation measures

Remember Rule 3 above, when you were thinking about the potential impact of your work? Keep that in mind as you think about how to validate your method. If you are producing, for example, a predictive model, then textbook measurements of predictive accuracy, such as the area under the receiver operating characteristic curve, may or may not matter to the end user of your system. Ask yourself what the end user cares about and what will make the work most impactful—whether it is increased statistical power, balancing different types of errors, or finding meaningful structure in data—and then design a rigorous, quantitative measure that captures this property. Although you should look at (and report) performance measures that have been used in prior work, you should not feel constrained to use only those measures if you don’t believe they accurately measure the impact of your approach.

### Rule 9: Set up version control

Use a modern version control system, such as git, and use it right away. Often, the start of a project is characterized by lots of writing, both of the writeup we’ve been discussing as well as code. You do not want to get several weeks into your project and then realize that you have no record of the initial versions of your code. If you are not familiar with version control, you should learn about it [4]. You should also give some thought to how to organize your repository to ensure reproducibility [5].

### Rule 10: Share your idea with colleagues

Once you have written out your idea, share it with trusted colleagues. The goal here is to elicit feedback about whether the idea makes sense and addresses an important, unsolved problem. If your writeup is short and compelling, then it’s not a big ask to have someone read over it and discuss it with you. You can also use this writeup as a way to recruit collaborators, if you need them, or to give trainees an overview of the project before they start working on it with you.

It is important to keep in mind that the order in which these rules are applied is not set in stone. For example, you may check for available data (Rule 7) before writing anything down (Rule 1). One of my goals here is to help you avoid unintentionally skipping over important steps, such as clearly defining the problem you want to solve or reading related work.

Once you have defined your computational biology project, you can start working on it. Alternatively, you can put it aside to work on another day. In general, a good habit to get into is to regularly produce short project descriptions like this whenever you have an exciting idea, even if you don’t have time to work on the idea right now. The process of producing the description can help clarify your level of excitement about the idea, and the writeup itself will ensure that you don’t forget the details. At the very least, if someone scoops you with a high-impact paper that recapitulates your idea, you’ll have the proof that you thought of it first.
